# Prognostic Significance of Sarcopenia in Patients Undergoing Surgery for Perihilar Cholangiocarcinoma: A Systematic Review and Meta-Analysis

**DOI:** 10.3390/cancers17050837

**Published:** 2025-02-28

**Authors:** Anastasia Efstathiou, Pablo Suarez Benitez, Shahin Hajibandeh, Shahab Hajibandeh, Thomas Satyadas

**Affiliations:** 1 Department of Surgery, University Hospital of Wales, Cardiff CF14 4XW, UK; anastasia.efstathiou@wales.nhs.uk; 2 Department of Surgery, Royal Gwent Hospital, Newport NP20 2UB, UK; pablo.suarezbenitez@wales.nhs.uk; 3 Department of Hepatobiliary and Pancreatic Surgery, Royal Stoke University Hospital, Stoke-on-Trent ST4 6QG, UK; 4 Department of Hepatobiliary and Pancreatic Surgery, Morriston Hospital, Swansea SA6 6NL, UK; shahab.hajibandeh@wales.nhs.uk; 5 Department of Hepatobiliary and Pancreatic Surgery, Manchester Royal Infirmary Hospital, Manchester M13 9WL, UK; thomas.satyadas@mft.nhs.uk

**Keywords:** sarcopenia, perihilar cholangiocarcinoma, liver resection

## Abstract

Aim: The aim of this study was to determine the impact of sarcopenia on outcomes in patients undergoing curative resection for perihilar cholangiocarcinoma. Methods: A systematic review and meta-analysis following PRISMA standards were conducted, searching for studies comparing patients with and without sarcopenia undergoing surgery for perihilar cholangiocarcinoma. The outcomes included postoperative mortality, Clavien–Dindo ≥ 3 complications, intraoperative blood loss, need for blood transfusion, length of hospital stay, and overall survival (OS) (time-to-event). The odds ratio (OR), mean difference (MD), and adjusted hazard ratio (HR) were calculated as summary measures using random effect modelling. Risk of bias was assessed with the Quality in Prognosis Studies tool. Results: Five studies featuring 1304 patients were included. There was no significant difference in postoperative mortality (OR 1.85, 95% CI 0.75–4.57, *p* = 0.18), Clavien–Dindo ≥ 3 complications (OR 1.44, 95% CI 0.92–2.25, *p* = 0.11), length of hospital stay (MD 2.13 days, 95% CI −0.89–5.15, *p* = 0.17) or OS (adjusted HR 1.48, 95% CI, 0.97–2.28, *p* = 0.07) between the patients with and without sarcopenia. Sarcopenia increased intraoperative blood loss (MD 388.00 mL, 95% CI, 114.99–683.01, *p* = 0.006) and the need for blood transfusion (OR 2.27, 95% CI, 1.66, 3.10, *p* < 0.00001). Conclusions: Sarcopenia may increase the risk of bleeding during the resection of perihilar cholangiocarcinoma (low certainty); however, this may not translate into a higher risk of postoperative morbidity or mortality (moderate certainty). Our findings regarding the OS may be subject to type 2 error; hence, the effect of sarcopenia on long-term outcomes after the resection of perihilar cholangiocarcinoma remains unknown and requires further research.

## Simple Summary

Perihilar cholangiocarcinoma is a cancer of bile ducts (tubes carrying bile from the liver to the bowel) around the liver, which is associated with a high risk of death, even with successful surgery. Sarcopenia is the loss of muscle mass due to ageing. This study investigated the impact of sarcopenia on outcomes of surgery for perihilar cholangiocarcinoma and found that, although sarcopenia may increase the risk of bleeding during surgery, it does not increase the risk of death or complications in patients with perihilar cholangiocarcinoma. However, the impact of sarcopenia on other long-term outcomes, such as overall survival, deserves further high-quality research, as currently available studies have not been able to provide strong and solid evidence in this context.

## 1. Introduction

Perihilar cholangiocarcinoma (PHCC), also known as Klatskin tumour, is a rare form of biliary cancer associated with poor prognosis [1], responsible for approximately 50–70% of all cholangiocarcinomas [2]. They are usually adenocarcinomas that arise from the biliary duct epithelium and occur at the hilar bifurcation of the hepatic ducts [2]. Klatskin tumours tend to be diagnosed late, as they present with non-specific symptoms until they reach advanced stages, leading to delayed diagnosis and poor survival rates [3,4]. The most common symptom is jaundice, which is present in 90% of cases [3]. This is followed by weight loss and abdominal pain in 35%, pruritus in 26%, and acute cholangitis in 10% [3].

Even though convoluted and challenging, the only plausible curative treatment is complete surgical resection, with only 30% of tumours being resectable at the time of diagnosis [4]. To achieve an R0 resection and avoid post-hepatectomy liver failure, an accurate preoperative evaluation of the tumour extension and liver status (i.e., volume, quality, and function of future liver remnant) is mandatory [3]. A right-sided tumour is best resected with an extended right hepatectomy and is likely to require portal vein resection. An advanced left-sided tumour is best resected with a left trisectionectomy and is likely to require portal vein and right hepatic arterial resections [3]. Neoadjuvant chemotherapy and/or radiotherapy for locally advanced PHCC does not seem to affect the oncological outcomes. However, they may allow tumour downstaging and improve tumour resectability [3].

While aiming to achieve negative margins to improve long-term survival, the complexity of the anatomical location and extent of tumour infiltration make biliary and hepatic resection a highly morbid procedure, with rates ranging from 14% to 76% [4]. This highlights the critical need for further research into this subtype of cancer to inform and optimise preoperative risk factors, ultimately leading to improved clinical outcomes. After curative resection, the 5-year survival rate ranges between 10 and 40% [4].

Sarcopenia is defined as the loss of skeletal muscle and its function [5]. It has been recognised as an irrefutable risk factor for poor outcomes, such as an increased risk of postoperative complications, an increased length of stay, and significantly lower survival rates across all surgical specialties [6,7]. The overall complication rate demonstrated across 160 different studies was 39.5% and 26.8% in patients with and without sarcopenia, respectively [7]. More specifically, as further demonstrated in a meta-analysis investigating the predictive value of sarcopenia in surgically treated cholangiocarcinoma cases, sarcopenia was recognised as a strong risk factor and predictor of poor postoperative outcomes [8].

The prognostic significance of sarcopenia, specifically in perihilar cholangiocarcinoma, remains widely unexplored, despite its established role in other cancer types. The aim of this review is to investigate the short-term and long-term outcomes of patients with sarcopenia undergoing curative surgery for perihilar cholangiocarcinoma, a subject which has yet to be systematically reviewed. This subject has been explored in some observational studies, providing a strong basis for synthesising evidence. Through exploring this relationship, we aim to fill a gap in the literature by conducting a comprehensive systematic review and meta-analysis of reported outcome measures from all available comparative studies to evaluate the impact of sarcopenia on outcomes of curative resection for perihilar cholangiocarcinoma.

## 2. Materials and Methods

### 2.1. Methodological and Reporting Compliance

This study was carried out and documented in accordance with the Preferred Reporting Items for Systematic reviews and Meta-Analyses (PRISMA) 2020 statement standards [9] and the Cochrane Handbook for Systematic review (version 6.4) [10].

### 2.2. Registration and Protocol

This study followed a predetermined protocol, as outlined and registered in PROSPERO, which is a publicly available international database for prospectively registered systematic reviews (PROSPERO registration number: CRD42025643576).

### 2.3. Eligibility Criteria

Study design: All comparative observational studies were regarded eligible for inclusion. Scoping reviews, literature reviews, case reports/series, single-arm studies, systematic reviews, and meta-analyses were excluded. Studies in which primary data were not available, such as conference abstracts, commentaries, correspondence articles, and editorials, were excluded.

Population: All patients aged 18 and above who underwent curative surgery for perihilar cholangiocarcinoma were eligible for inclusion.

Prognostic factor: Sarcopenia identified using CT-measured psoas or skeletal muscle mass index was the studied prognostic factor. Patients with sarcopenia were compared with those without sarcopenia.

Outcomes: The outcomes of interest included postoperative mortality, complication rate defined by a Clavien–Dindo [11] score over or equal to 3, intraoperative blood loss, need for blood transfusion, length of hospital stay, and overall survival (OS) (time-to-event).

### 2.4. Information Sources and Search Strategy

A thorough search strategy was generated, comprising keywords, thesaurus headings, limits, and Boolean operators adjusted for each database. The databases used included Scopus^®^, MEDLINE^®^, Embase^®^, and PubMed^®^. Two authors conducted the search independently. The results of the two searches were then reviewed by a third independent author to reach a consensus in the event of disparities between the selected articles. The search was completed on the 5 January 2025, with no language constraints. In addition to the above, reference lists of the identified articles and previous systematic reviews were checked to identify additional potentially eligible articles.

### 2.5. Study Selection, Data Collection, and Data Items

Subsequent to the implementation of the search strategy and the removal of duplicate articles, the remaining potentially eligible articles were screened by two authors independently. The screening process consisted of a review of the article’s title and abstract against the predetermined eligibility criteria and, thereafter, full-text examination to identify eligible articles. Articles in line with the eligibility criteria were selected for inclusion. Following the identification of relevant eligible articles by each author independently, a third author reviewed the data independently and provided an opinion regarding eligibility. The data items of interest were determined amidst formulation of the protocol by authors expert in evidence synthesis, following eligible study selection, through the utilisation of a pilot-testing technique for randomly selected studies. The data items were collated in an electronic data sheet by two authors independently. These consisted of bibliometric parameter information, patient demographics, including population, sample size, definition of sarcopenia, interventions such as neoadjuvant chemotherapy, preoperative biliary drainage, portal vein embolisation, Bismuth classification of disease, type and duration of operation, tumour, lymph node and resection status, estimated blood loss, need for blood transfusion, postoperative mortality, complications (Clavien–Dindo ≥ 3), length of hospital stay, and adjusted hazard ratio (HR) of OS.

### 2.6. Study Risk of Bias and Evidence Certainty Assessment

The Quality In Prognosis Studies (QUIPS) tool was implemented to assess the risk of bias in study participation, study attrition, measurement of prognostic factor, measurement of outcomes, study confounding, and statistical analysis [12]. The GRADE (Grading of Recommendations Assessment, Development and Evaluation) system was used for assessing the certainty of evidence [13].

### 2.7. Effect Measures and Synthesis Methods

RevMan Web was used for the analyses. The odds ratio (OR) was calculated as a summary effect measure for dichotomous variables, and the mean difference (MD) was calculated for continuous variables. The OS was analysed as a time-to-event outcome by calculating the adjusted HR using the generic inverse variance method in order to address the uncertainties associated with varying follow-up periods among the included studies. Random-effect modelling was used for the analyses, and forest plots with 95% confidence intervals (CIs) were constructed to present the results. Individual patients were considered as a unit of analysis. Statistical heterogeneity was measured as I^2^ using Cochran’s Q test (χ^2^), and heterogeneity was classified as low when I^2^ was 0–25%, moderate when I^2^ was 25–75%, and high when I^2^ was 75–100%.

### 2.8. Reporting Bias Assessment

According to the formulated protocol, the risk of reporting bias would be examined by assembling a funnel plot if the outcomes were described by at least 10 studies; however, since the outcomes were described by less than 10 studies, the reporting bias evaluation was not feasible.

### 2.9. Deviation from the Registered Protocol

There was no deviation from the registered protocol.

## 3. Results

### 3.1. Study Selection and Characteristics

**Figure 1:**
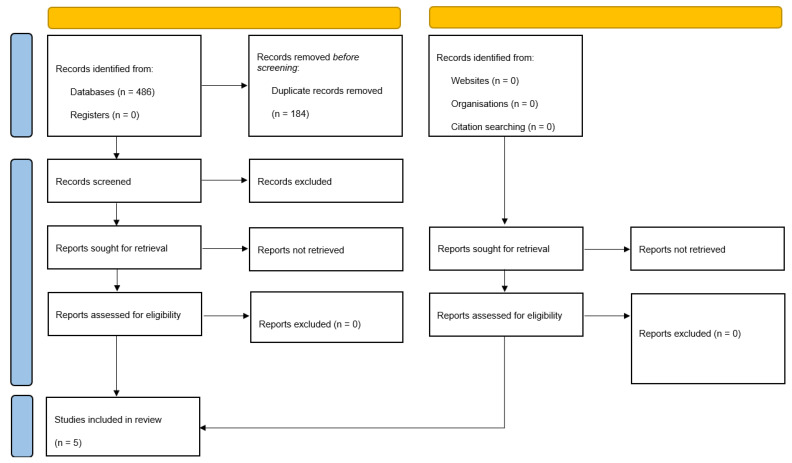
Study PRISMA flow diagram.

The search strategy for the databases delineated above yielded a total of 486 studies (Figure 1, PRISMA flow diagram); 302 remained following the elimination of duplicates. Proceeding with the screening of the titles and abstracts of the remaining studies, 297 articles did not meet the principal eligibility criteria and were excluded directly; therefore, a total of 5 potentially eligible articles were identified. After reviewing the full-text articles, suitability was confirmed, leading to five retrospective observational studies [14,15,16,17,18] including a sample of 1304 patients with a diagnosis of perihilar cholangiocarcinoma which underwent surgical resection. Of those, 482 patients were classed as sarcopenic and 822 as non-sarcopenic based on CT-measured psoas/skeletal muscle mass index (Table 1 and Table 2).

### 3.2. Risk of Bias

The risk of bias assessment using the QUIPS tool showed a low risk of bias in the following domains: study participation, study attrition, prognostic factor measurement, outcome measurement, and statistical analysis. The risk of bias due to study confounding was low in two studies and unclear in three studies (Supplementary Table S1).

### 3.3. Comparative Analysis of Operative Outcomes

#### 3.3.1. Postoperative Mortality

**Figure 2:**
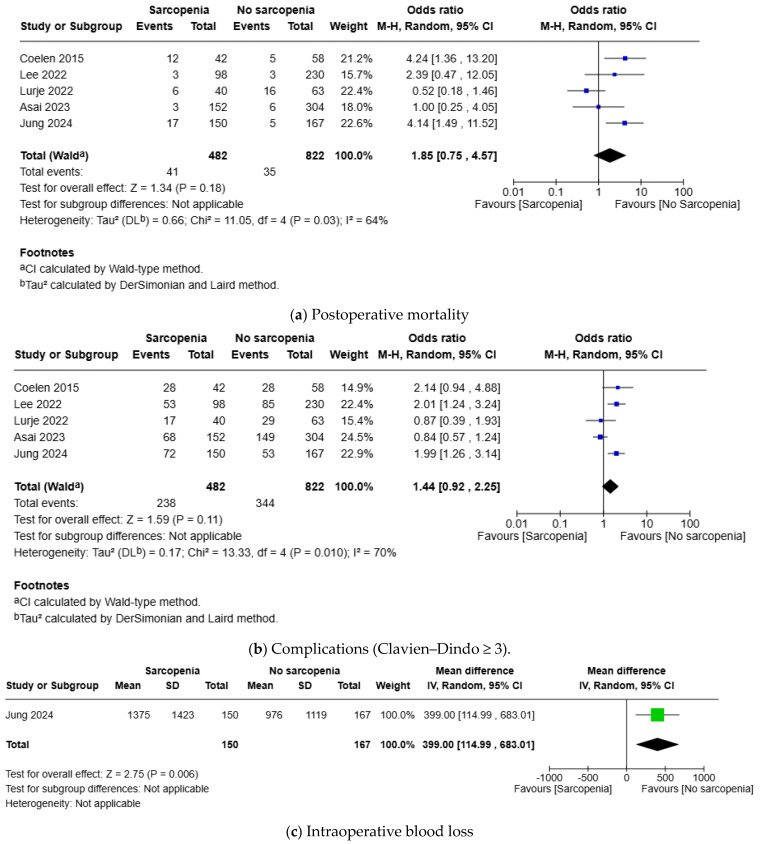

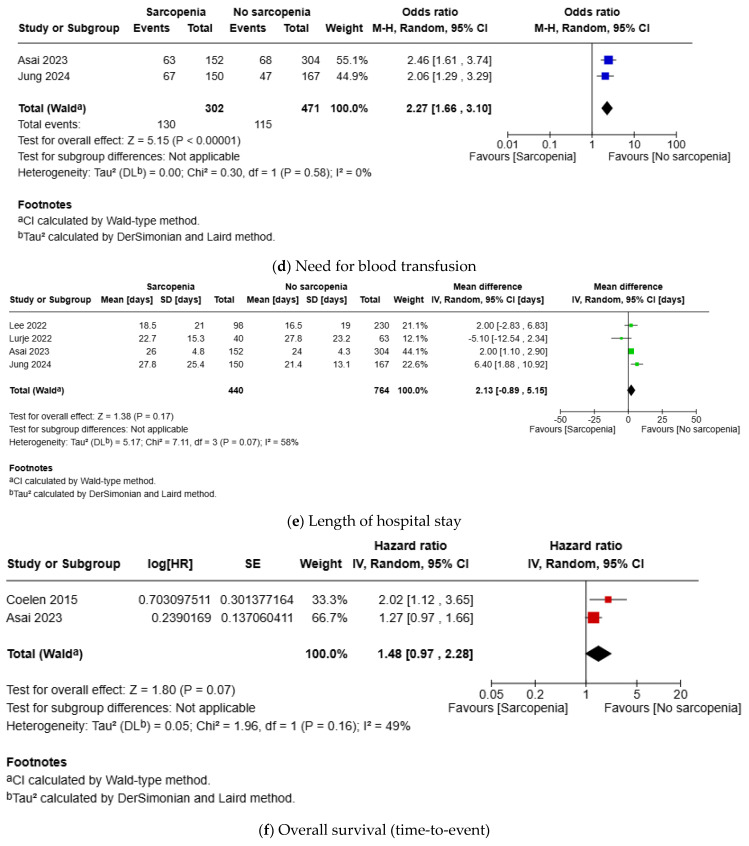
Forest plots for the comparison of outcomes between the groups with and without sarcopenia: (**a**) postoperative mortality; (**b**) complications (Clavien–Dindo ≥ 3); (**c**) intraoperative blood loss; (**d**) need for blood transfusion; (**e**) length of hospital stay; and (**f**) overall survival (time-to-event). Included studies: Jung, 2024 [14], Asai, 2023 [15], Lee, 2022 [16], Lurje, 2022 [17], and Coelen, 2015 [18].

Postoperative mortality was reported in five studies including a sample size of 1304 patients; there was no difference in the risk of postoperative mortality between patients with sarcopenia and those with no sarcopenia (OR 1.85, 95% CI 0.75, 4.57, *p* = 0.18) (Figure 2). The level of heterogeneity among the included studies was found to be moderate (I^2^ = 64%, *p* = 0.03), and the certainty of evidence was moderate.

**Table 2:** Baseline characteristics of the included population.

Study	Age *#	Male Sex *	BMI *#	Preoperative Biliary Drainage *	Neoadjuvant Chemotherapy *	Portal Vein Embolisation *	Bismuth Classification *				T Stage *		N Stage *		R0 Resection *	Operation Time (min) *#
							I	II	III	IV	0–2	3–4	0	1–2		
Jung, 2024 [14]	66.5 ± 8.2 vs. 64.7 ± 9.5	106/150 vs. 96/167	22.96 ± 2.75 vs. 23.83 ± 2.58	NR	19/150 vs. 8/167	43/150 vs. 53/167	5/150 vs. 5/167	14/150 vs. 26/167	88/150 vs. 92/167	43/150 vs. 44/167	114/148 vs. 139/166	34/148 vs. 27/166	99/150 vs. 110/167	51/150 vs. 57/167	116/150 vs. 118/167	550.7 ± 172.7 vs. 506.7 ± 169.7
Asai, 2023 [15]	72 (65–77) vs. 68 (61–73)	103/152 vs. 205/304	NR	135/152 vs. 264/304	NR	NR	NR	NR	NR	NR	NR	NR	NR	NR	NR	555 (482–644) vs. 544 (485–624)
Lee, 2022 [16]	66.2 (46–82) vs. 64.5 (42–89)	75/98 vs. 145/230	24.6 (17.4–33.5) vs. 22.8 (14.8–40.5)	NR	0/98 vs. 5/230	13/98 vs. 40/230	0/98 vs. 0/230	0/98 vs. 0/230	98/98 vs. 230/230	0/98 vs. 0/230	85/97 vs. 195/226	12/97 vs. 31/226	75/98 vs. 147/230	23/98 vs. 83/230	90/98 vs. 198/230	420.8 (194–1400) vs. 394.7 (180–965)
Lurje, 2022 [17]	NR	NR	NR	NR	0/40 vs. 0/63	NR	NR	NR	NR	NR	NR	NR	NR	NR	NR	NR
Coelen, 2015 [18]	61 ± 11 vs. 62 ± 9	28/42 vs. 36/58	24 ± 3 vs. 26 ± 3	NR	NR	NR	0/42 vs. 1/58	2/42 vs. 4/58	27/42 vs. 33/58	9/42 vs. 12/58	19/42 vs. 24/58	23/42 vs. 34/58	30/42 vs. 45/58	12/42 vs. 13/58	32/42 vs. 40/58	NR

* Sarcopenia group vs. non sarcopenia group. # Mean ± standard deviation or median (range). NR: not reported; BMI: body mass index.

#### 3.3.2. Complications (Clavien–Dindo ≥ 3)

Clavien–Dindo ≥ 3 complication was reported in five studies including a sample of size 1304. There was no difference in the risk of Clavien–Dindo ≥ 3 complications between patients with sarcopenia and those with no sarcopenia (OR 1.44, 95% CI 0.92, 2.25, *p* = 0.11) (Figure 2). The level of heterogeneity among the included studies was found to be moderate (I^2^ = 70%, *p* = 0.01), and the certainty of evidence was moderate.

#### 3.3.3. Intraoperative Blood Loss

Intraoperative blood loss was reported in one study with a sample of 317 patients. Intraoperative blood loss was higher in patients with sarcopenia (MD 388.00 mL, 95% CI 114.99, 683.01, *p* = 0.006) (Figure 2). Heterogeneity was not applicable as only one study included this analysis, and the certainty of evidence was low.

#### 3.3.4. Need for Blood Transfusion

Need for blood transfusion was reported in two studies featuring a sample of size of 773 patients. The need for blood transfusion was higher in patients with sarcopenia (OR 2.27, 95% CI 1.66, 3.10, *p* < 0.00001) (Figure 2). The level of heterogeneity among the included studies was found to be low (I^2^ = 0%, *p* = 0.58), and the certainty of evidence was moderate.

#### 3.3.5. Length of Hospital Stay

The length of hospital stay was reported in four studies including a sample of 1204 patients. There was no difference in the length of hospital stay between the patients with and without sarcopenia (MD 2.13 days, 95% CI −0.89, 5.15, *p* = 0.17) (Figure 2). The level of heterogeneity among the included studies was found to be moderate (I^2^ = 58%, *p* = 0.07), and the certainty of evidence was moderate.

#### 3.3.6. Overall Survival (Time-to-Event)

The OS was reported in two studies including a sample of 556 patients. There was no difference in the OS between the groups with and without sarcopenia (adjusted HR: 1.48, 95% CI 0.97, 2.28, *p* = 0.07). The level of heterogeneity among the included studies was found to be moderate (I^2^ = 49%, *p* = 0.16), and the certainty of evidence was low.

## 4. Discussion

We conducted a systematic review and meta-analysis to compare the outcomes of patients with and without sarcopenia undergoing curative resection for perihilar cholangiocarcinoma. The analysis of 1304 patients from five studies showed no difference in postoperative mortality (moderate certainty), Clavien–Dindo ≥ 3 complications (moderate certainty), length of hospital stay (moderate certainty), and OS (low certainty) between the patients with and without sarcopenia; however, sarcopenia increased intraoperative blood loss (low certainty) and the need for blood transfusion (moderate certainty). The certainty was mainly downgraded due to between-study heterogeneity and the small number of included studies.

Although the prognostic significance of sarcopenia in patients with cholangiocarcinomas has been studied in previous systematic reviews, this study is the first systematic review and meta-analysis that explores the effect of sarcopenia specifically in patients with perihilar cholangiocarcinoma undergoing curative operation. He et al. [8] conducted a systematic review of 33 articles including all types of cholangiocarcinomas and concluded that sarcopenia had a negative impact on postoperative complications and survival outcomes. In another systematic review, Surov et al. [19] examined 16 studies including all types of cholangiocarcinomas and concluded that sarcopenia had a negative impact on the survival outcomes. The results of the current study are not consistent with the findings of the above studies. The difference between the findings of the current study and previous studies can be explained as follows. Firstly, the previous studies included all cases of cholangiocarcinomas, while the current study included patients with perihilar cholangiocarcinoma. It is well recognised that perihilar, distal, and intrahepatic cholangiocarcinomas vary in their presentation, natural history, and therapeutic approach to their management [20]; hence, the difference in the inclusion criteria between the current study and previous systematic review may explain the difference in the findings. Moreover, in the current study, the OS was reported only by two studies, and the finding of no difference between the two groups could have been simply due to a type 2 error; therefore, more studies are required to provide robust conclusions on effect of sarcopenia on the OS in patients with perihilar cholangiocarcinoma.

The results of the current study suggested that sarcopenia may increase the risk of intraoperative bleeding and the need for blood transfusion. Patients with sarcopenia have reduced skeletal muscle capillary density [21]. On the other hand, patients with sarcopenia may have a reduced total blood volume compared to those without [22]. Consequently, patients with sarcopenia may experience increased proportional blood loss requiring blood transfusion during surgery [22]. Considering these findings, we recommend the careful management of blood products in such patients with cancer to avoid debilitating transfusion-related complications. Nevertheless, our results showed that increased intraoperative bleeding may not translate into increased postoperative morbidity and mortality.

We came across some limitations during the elaboration of this study. Namely, there was a limited number of studies in the available literature specifically delving into the impact of sarcopenia, both in the short and long term, on surgical outcomes for perihilar cholangiocarcinoma. This translated into a small sample size and the risk of type 2 error in the analysis of some of the outcomes, such as the OS. Furthermore, given the retrospective nature of the included studies, there was an unavoidable inherent risk of selection bias. The between-study heterogeneity was moderate, and the risk of confounding bias was unclear in three of the studies; therefore, we had to downgrade the certainty of the available evidence accordingly. Publication bias could not be assessed due to the small number of included studies. Moreover, although the baseline characteristics of the included patients were well reported by the included studies, the data on the nutritional care of such patients were poorly reported. Considering the importance of nutrition in patients with cancer, underscoring its potential to improve survival, quality of life, and overall treatment success [23], this might have subjected the findings of this study to some bias. Finally, the diagnostic process to which the study populations were subjected in each study before the decision made during the multidisciplinary meeting was not clear.

## 5. Conclusions

Sarcopenia may increase the risk of bleeding during the resection of perihilar cholangiocarcinoma (low certainty); however, this may not translate into a higher risk of postoperative morbidity or mortality (moderate certainty). Our findings regarding the OS may be subject to type 2 error; hence, the effect of sarcopenia on long-term outcomes after the resection of perihilar cholangiocarcinoma remains unknown and requires further research.

## Figures and Tables

**Table 1:** Data related to the included studies.

First Author	Year	Country	Journal	Design	Included Population	Sample size			Definition of Sarcopenia
						Total	Sarcopenia	No Sarcopenia	
Jung [14]	2024	Korea	*Front. Oncol.*	Retrospective observational	Patients undergoing hepatic resection for perihilar cholangiocarcinoma	317	150	167	CT-measured psoas muscle mass index
Asai [15]	2023	Japan	*J. Hepatobiliary Pancreat. Sci.*	Retrospective observational	Patients undergoing hepatic resection for perihilar cholangiocarcinoma	456	152	304	CT-measured psoas muscle mass index
Lee [16]	2022	Korea	*J. Clin. Med.*	Retrospective observational	Patients undergoing hepatic resection for perihilar cholangiocarcinoma	328	98	230	CT-measured skeletal muscle index
Lurje [17]	2022	Germany	*Hepatol. Commun.*	Retrospective observational	Patients undergoing hepatic resection for perihilar cholangiocarcinoma	103	40	63	CT-measured skeletal muscle index
Coelen [18]	2015	Netherlands	*HPB*	Retrospective observational	Patients undergoing hepatic resection for perihilar cholangiocarcinoma	100	42	58	CT-measured skeletal muscle index

CT: computed tomography.

## Supplementary Materials

The following supporting information can be downloaded at https://www.mdpi.com/article/10.3390/cancers17050837/s1, Supplementary Table S1: Results of risk of bias.
