# Supplementary material for: Prognostic Significance of Sarcopenia in Patients Undergoing Surgery for Perihilar Cholangiocarcinoma: A Systematic Review and Meta-Analysis
Source: Cancers (Basel). 2025 Feb 28;17(5):837. doi: 10.3390/cancers17050837 (PMC11899633; doi:10.3390/cancers17050837)
Supplement: Supplementary file 1 [file cancers-17-00837-s001.zip › cancers-3484012-supplementary.pdf]

Supplementary Table 1. Results of risk of bias of the included case reports using the QUIPS (Quality In Prognosis Studies) tool							
**First Author**	**Year**	**Study participation**	**Study attrition**	**Prognostic factor measurement**	**Outcome measurement**	**Study confounding**	**Statistical analysis**
Jung	2024	Low risk of bias	Low risk of bias	Low risk of bias	Low risk of bias	Low risk of bias	Low risk of bias
Asai	2023	Low risk of bias	Low risk of bias	Low risk of bias	Low risk of bias	Unclear risk of bias	Low risk of bias
Lee	2022	Low risk of bias	Low risk of bias	Low risk of bias	Low risk of bias	Low risk of bias	Low risk of bias
Lurje	2022	Low risk of bias	Low risk of bias	Low risk of bias	Low risk of bias	Unclear risk of bias	Low risk of bias
Coelen	2015	Low risk of bias	Low risk of bias	Low risk of bias	Low risk of bias	Unclear risk of bias	Low risk of bias
